# Synergistic Anticancer Effect of a Combination of Berbamine and Arcyriaflavin A against Glioblastoma Stem-like Cells

**DOI:** 10.3390/molecules27227968

**Published:** 2022-11-17

**Authors:** Jang Mi Han, Hye Jin Jung

**Affiliations:** 1 Department of Life Science and Biochemical Engineering, Graduate School, Sun Moon University, Asan 31460, Republic of Korea; 2 Department of Pharmaceutical Engineering and Biotechnology, Sun Moon University, Asan 31460, Republic of Korea; 3 Genome-Based BioIT Convergence Institute, Sun Moon University, Asan 31460, Republic of Korea

**Keywords:** glioblastoma stem-like cells, berbamine, arcyriaflavin A, calcium/calmodulin-dependent protein kinase II gamma, cyclin-dependent kinase 4

## Abstract

Glioblastoma multiforme (GBM) is the most aggressive form of brain tumor. Relapse is frequent and rapid due to glioblastoma stem-like cells (GSCs) that induce tumor initiation, drug resistance, high cancer invasion, immune evasion, and recurrence. Therefore, suppression of GSCs is a powerful therapeutic approach for GBM treatment. Natural compounds berbamine and arcyriaflavin A (ArcA) are known to possess anticancer activity by targeting calcium/calmodulin-dependent protein kinase II gamma (CaMKIIγ) and cyclin-dependent kinase 4 (CDK4), respectively. In this study, we evaluated the effects of concurrent treatment with both compounds on GSCs. Combined treatment with berbamine and ArcA synergistically inhibited cell viability and tumorsphere formation in U87MG- and C6-drived GSCs. Furthermore, simultaneous administration of both compounds potently inhibited tumor growth in a U87MG GSC-grafted chick embryo chorioallantoic membrane (CAM) model. Notably, the synergistic anticancer effect of berbamine and ArcA on GSC growth is associated with the promotion of reactive oxygen species (ROS)- and calcium-dependent apoptosis via strong activation of the p53-mediated caspase cascade. Moreover, co-treatment with both compounds significantly reduced the expression levels of key GSC markers, including CD133, integrin α6, aldehyde dehydrogenase 1A1 (ALDH1A1), Nanog, Sox2, and Oct4. The combined effect of berbamine and ArcA on GSC growth also resulted in downregulation of cell cycle regulatory proteins, such as cyclins and CDKs, by potent inactivation of the CaMKIIγ-mediated STAT3/AKT/ERK1/2 signaling pathway. In addition, a genetic knockdown study using small interfering RNAs (siRNAs) targeting either CaMKIIγ or CDK4 demonstrated that the synergistic anticancer effect of the two compounds on GSCs resulted from dual inhibition of CaMKIIγ and CDK4. Collectively, our findings suggest that a novel combination therapy involving berbamine and ArcA could effectively eradicate GSCs.

## 1. Introduction

Glioblastoma multiforme (GBM) is the most aggressive and common form of malignant brain tumor in adults and has a poor prognosis [1]. The standard therapy for patients with GBM includes surgical resection followed by adjuvant radiotherapy and chemotherapy with the alkylating agent temozolomide (TMZ) [2]. Despite recent advances in disease treatment, the median survival of patients with GBM is estimated to be 12–15 months [3,4]. This limited survival rate is due to drug resistance and subsequent recurrence of GBM following chemotherapy and other treatments [3,5]. Although the mechanisms of GBM resistance are not yet fully understood, accumulating evidence has revealed that glioblastoma stem-like cells (GSCs) contribute to poor prognosis in patients with GBM [6]. GSCs are a subpopulation of GBM tumor cells that possess self-renewal and multi-lineage differentiation capacities and play important roles in tumor initiation and propagation, cancer invasion, immune evasion, treatment resistance, and tumor recurrence [7,8]. Therefore, targeting GSCs is considered a powerful therapeutic approach for GBM.

Combining drugs with different mechanisms of action can enhance cancer treatment effects and overcome drug resistance compared to single drug administration [9]. However, although combination therapy with the antiangiogenic agent bevacizumab and TMZ prolonged progression-free survival in patients with GBM, it did not increase overall survival due to tumor recurrence within 1–2 years in a phase III trial [10,11]. Therefore, discovering new drug combinations to effectively eradicate GSCs may be a promising strategy for improving the outcome of GBM treatment.

**Figure 1:**
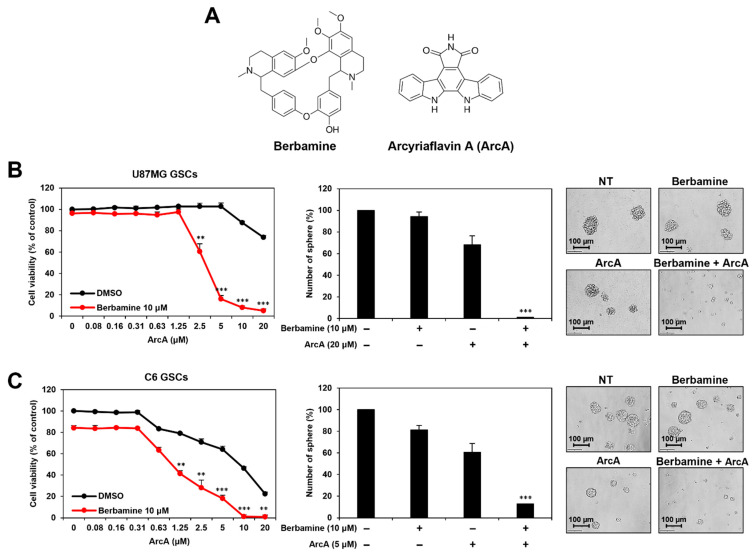
Combined treatment of berbamine and ArcA synergistically suppresses GSCs viability. (**A**) The chemical structures of berbamine and ArcA. (**B**,**C**) U87MG- and C6-derived GSCs were treated with the indicated concentrations of berbamine and ArcA for 7 days. Cell viability was measured using the CellTiter-Glo^®^ luminescent assay system. The number of formed tumorspheres was counted under an optical microscope. ** *p* < 0.01, *** *p* < 0.001 vs. the compound alone.

Berbamine is a natural bis-benzylisoquinoline alkaloid and the major bioactive compound isolated from traditional Chinese herbal medicine *Berberis amurensis* [12] (Figure 1A). Berbamine is known to possess various pharmacological properties, including antioxidant, anti-inflammatory, antihypertensive, antiarrhythmic, and antiangiogenic activities [13,14,15,16]. Several recent studies have demonstrated the antitumor effects of berbamine in various cancers [17,18,19,20,21,22,23,24,25,26,27]. Berbamine inhibits the proliferation of lung cancer, myeloma, prostate cancer, and liver cancer cells by activating the intrinsic pathway of apoptosis [17,18,19,20]. It also causes regression of GBM tumor progression by inhibiting angiogenesis [16]. Furthermore, berbamine potentiated the inhibitory effects of gemcitabine and paclitaxel on the growth of pancreatic and gastric cancer, respectively [21,22]. Berbamine has also been shown to suppress the growth of cancer stem cells (CSCs) by targeting calcium/calmodulin-dependent protein kinase II gamma (CaMKIIγ) [23,24,25]. Berbamine inhibits the self-renewal abilities of leukemia stem cells and liver CSCs by inhibiting kinase activity through binding specificity for the ATP-binding pocket of CaMKIIγ [23,24]. More recently, it was found that combined treatment with CaMKII inhibitors, including berbamine, hydrazinobenzoylcurcumin (HBC), and KN93, and neurokinin 1 receptor (NK1R) inhibitors, such as SR 140,333 and aprepitant, increased GSC lethality [25]. The synthetic lethal interaction between CaMKIIγ and NK1R in GSCs has been demonstrated by gene silencing using small interfering RNAs (siRNAs), suggesting a new combination therapy targeting CaMKIIγ and NK1R to eliminate GSCs [25]. Therefore, CaMKIIγ is an attractive anticancer target for combating CSCs, including GSCs, and the discovery of novel compounds that can synergistically increase the suppressive effects of berbamine may provide another promising CaMKII-targeted combination therapy for the effective treatment of GBM.

In proliferating cells, cyclin-dependent kinase 4/6 (CDK4/6) binds to cyclin D1, and the complex subsequently phosphorylates retinoblastoma (Rb) to release the transcription factor E2F, which then drives cell cycle progression [28,29]. In several cancers, including GBM, the CDK4/6-cyclin D-Rb-E2F pathway is excessively activated to promote cancer cell proliferation [30,31]. Therefore, targeting the cell cycle pathway is a rational option for cancer treatment [31]. CDK4/6 inhibitors, such as palbociclib, ribociclib, and abemaciclib, have been widely used in preclinical and clinical trials as anticancer drugs [32]. They suppress proliferation and induce apoptosis in a variety of tumor cells, including GBM, by inhibiting the CDK4/6-cyclin D-Rb-E2F pathway [31,32,33,34,35]. However, intrinsic or acquired resistance to CDK4/6 inhibitors has limited their application in cancer therapy [36]. Therefore, the discovery of new CDK4/6 inhibitors and the development of effective drug combination strategies to overcome this resistance are urgently required. Arcyriaflavin A (ArcA), a natural compound found in myxomycetes *Arcyria obvelata* and *Arcyria denudata*, inhibits CDK4 and CaMKII [37] (Figure 1A). ArcA inhibited the replication of human cytomegalovirus, suppressed proliferation, and induced apoptosis of human colon cancer, lung cancer, and endometriotic stromal cells [38,39,40]. However, the anticancer activity and underlying molecular mechanisms of ArcA in GBM have not yet been studied.

In the present study, we demonstrated for the first time the synergistic anticancer effect of a combination of berbamine and ArcA on GSCs in vitro and in vivo. We also found that increased inhibitory activity in the growth of GSCs by the combination treatment was associated with the dual inhibition of CaMKIIγ and CDK4. Thus, these findings suggest a novel CaMKIIγ-targeted combination therapy involving berbamine and ArcA to eradicate GSCs.

## 2. Results

### 2.1. Combined Treatment of Berbamine and ArcA Synergistically Suppresses GSC Viability

To explore novel combination therapies for efficient suppression of GSCs, we previously performed high-throughput drug combination screening using CaMKII inhibitors including berbamine and a bioactive compound library [25]. As a newly discovered drug combination, the potent CDK4 inhibitor ArcA and berbamine synergistically increased the lethality of U87MG- and C6-derived GSCs. As shown in Figure 1B,C and Figure S1, co-treatment with berbamine and ArcA significantly inhibited the viability and tumorsphere formation of U87MG- and C6-derived GSCs compared to single-compound treatments. These results suggest that combined treatment with berbamine and ArcA has a promising anticancer effect in effectively eliminating GSCs.

### 2.2. Combined Treatment of Berbamine and ArcA Strongly Promotes GSC Apoptosis

**Figure 2:**
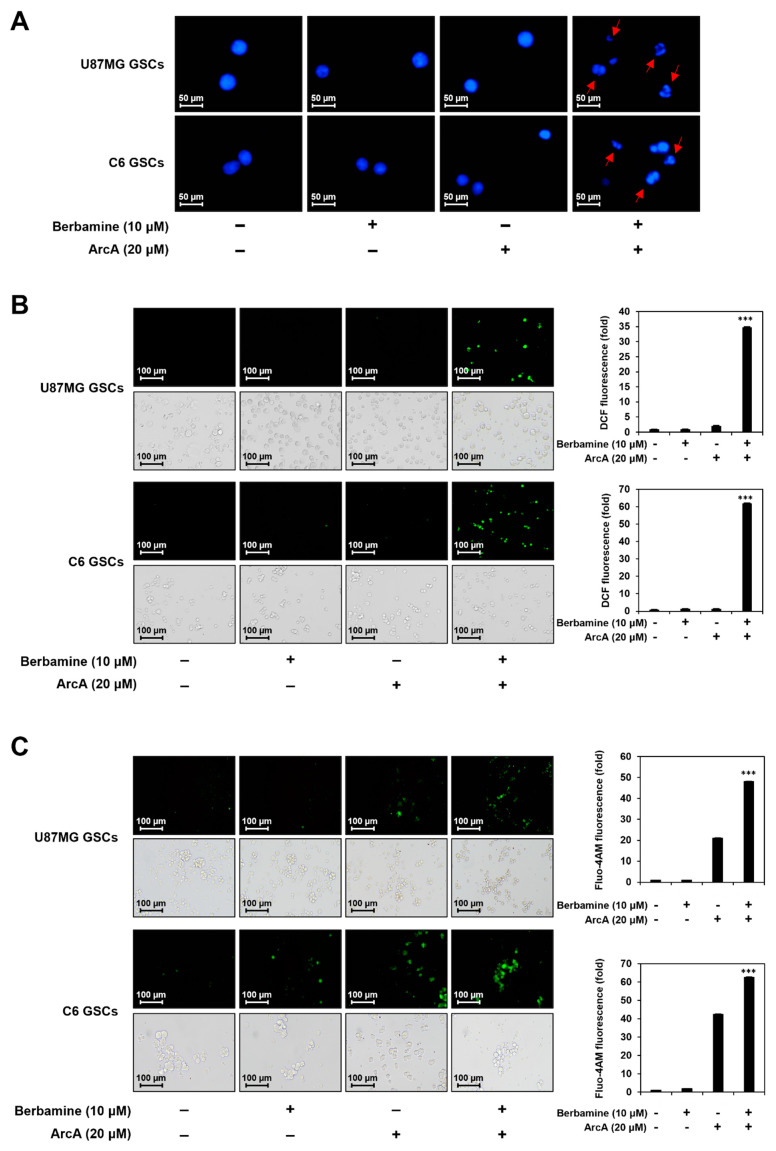

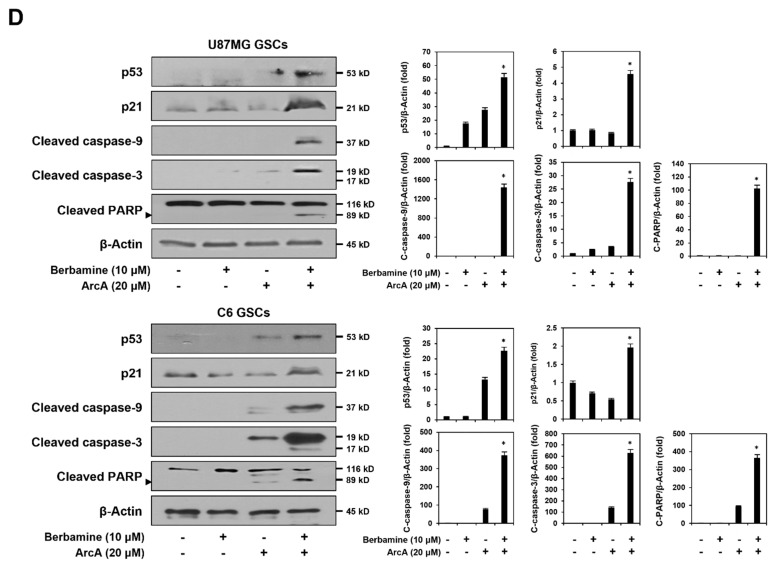
Combined treatment of berbamine and ArcA strongly promotes GSC apoptosis. (**A**–**D**) U87MG- and C6-derived GSCs were treated with the indicated concentrations of berbamine and ArcA for 24 h. (**A**) Effect of combined treatment of berbamine and ArcA on the nuclear morphology. Changes in nuclear morphology were monitored by DAPI staining under a fluorescence microscope. (**B**) Effect of combined treatment of berbamine and ArcA on the intracellular ROS generation. ROS levels were detected with H_2_DCFDA using a fluorescence microscope and were further quantified by densitometry. The level of DCF fluorescence for untreated control was normalized to 1-fold. (**C**) Effect of combined treatment of berbamine and ArcA on the intracellular calcium level. The levels of calcium were detected with Fluo-4 AM using a fluorescence microscope and were further quantified by densitometry. The level of Fluo-4 AM fluorescence for untreated control was normalized to 1-fold. (**D**) Effect of combined treatment of berbamine and ArcA on the expression of apoptosis regulators. Protein levels were detected by Western blot analysis using specific antibodies and were further quantified by densitometry. β-Actin levels were used as an internal control. The ratio of each target protein to β-actin for untreated control was normalized to 1-fold. * *p* < 0.05, *** *p* < 0.001 vs. the compound alone.

Next, we investigated whether the combined effect of berbamine and ArcA in inhibiting GSC viability is associated with the promotion of apoptosis. As shown in Figure 2A, co-treatment with berbamine and ArcA for 24 h strongly induced the nuclear condensation and fragmentation of U87MG- and C6-derived GSCs.

The generation of reactive oxygen species (ROS) is closely related to the induction of apoptosis mediated by mitochondria and the endoplasmic reticulum (ER) [41]. As shown in Figure 2B, combined treatment with berbamine and ArcA for 24 h markedly increased the intracellular ROS levels in U87MG- and C6-derived GSCs.

An increase in cytosolic calcium concentration can trigger the intrinsic apoptosis pathway by promoting the release of pro-apoptotic factors such as cytochrome c from the mitochondria and subsequent caspase activation [42,43]. Thus, we investigated whether combined treatment with berbamine and ArcA affected the intracellular calcium levels of GSCs. As shown in Figure 2C, co-treatment with berbamine and ArcA for 24 h increased the intracellular calcium concentration more than single-compound treatment in U87MG- and C6-derived GSCs.

The p53 tumor suppressor triggers the activation of the caspase cascade through the induction of specific apoptotic target genes [44]. We further evaluated the effect of simultaneous treatment with berbamine and ArcA on the expression of the major mediators of apoptosis in U87MG- and C6-derived GSCs. As shown in Figure 2D, co-treatment with the two compounds significantly increased p53 and its downstream effector p21 expression compared with single-compound treatments in both types of GSCs. The combination of berbamine and ArcA markedly upregulated the levels of cleaved caspase-9, caspase-3, and poly (ADP-ribose) polymerase (PARP). Taken together, these data demonstrate that the increase in GSC lethality induced by the combination of berbamine and ArcA resulted from the strong promotion of apoptosis through the synergistic activation of the ROS- and calcium-mediated caspase cascade in GSCs.

### 2.3. Combined Treatment of Berbamine and ArcA Potently Downregulates CaMKIIγ-Mediated Growth Signaling Pathway

**Figure 3:**
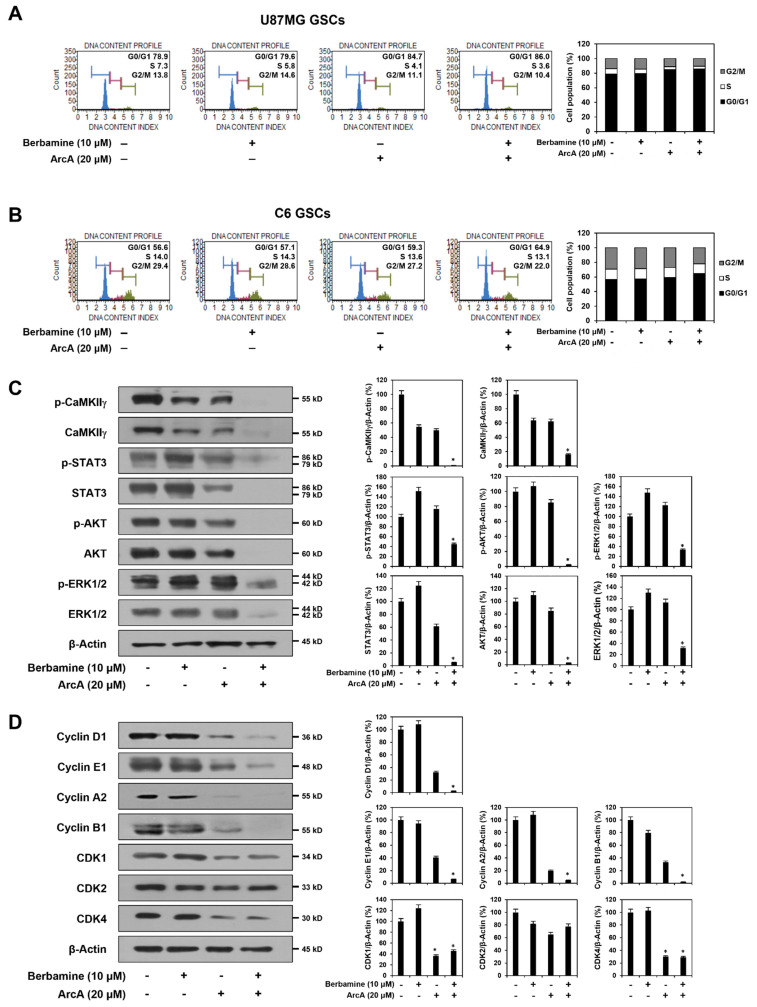
Combined treatment of berbamine and ArcA potently downregulates CaMKIIγ-mediated growth signaling pathway. (**A**–**D**) U87MG- and C6-derived GSCs were treated with the indicated concentrations of berbamine and ArcA for 24 h. (**A**,**B**) Effect of combined treatment of berbamine and ArcA on the cell cycle in both GSCs. Cell cycle distribution was detected using a Muse Cell Analyzer with Muse^®^ Cell Cycle kit. (**C**) Effect of combined treatment of berbamine and ArcA on the CaMKIIγ-mediated STAT3/AKT/ERK1/2 signaling pathway in U87MG-derived GSCs. (**D**) Effect of combined treatment of berbamine and ArcA on the expression of cell cycle regulatory proteins in U87MG-derived GSCs. (**C**,**D**) Protein levels were detected by Western blot analysis using specific antibodies and were further quantified by densitometry. β-Actin levels were used as an internal control. * *p* < 0.05 vs. the compound alone or the control.

CaMKII plays a significant role in the control of cell cycle machinery through the regulation of cyclins and CDKs and consequently induces cell proliferation [45,46]. Therefore, we further investigated whether the synergistic anticancer effect of the combination of berbamine and ArcA in GSCs was related to the modulation of CaMKIIγ-mediated cell cycle machinery. We first evaluated the effect of combined treatment with berbamine and ArcA on the proliferation of U87MG- and C6-derived GSCs using the ATP-monitoring luminescence assay. As shown in Figure S2, the proliferation of untreated control cells increased in a time-dependent manner. However, co-treatment of berbamine and ArcA more strongly inhibited the proliferation of both GSCs compared to single-compound treatments at the indicated time points. Next, compared to untreated control cells, co-treatment with both compounds for 24 h increased the G0/G1 phase cell population in U87MG- and C6-derived GSCs (Figure 3A,B). In addition, similar to the results in the 24 h treatment, combined treatment with the two compounds for the early time points, including 4, 8, and 12 h, increased the G0/G1 phase cell population, but not those at the S and G2/M phases, compared to single-compound treatments in both types of GSCs (Figure S3). These results indicate that the combined action of these compounds caused cell cycle arrest at the G0/G1 phase. Notably, combined treatment with berbamine and ArcA potently decreased both the total and phosphorylated protein levels of CaMKIIγ compared to single-compound treatments in U87MG-derived GSCs (Figure 3C). In addition, simultaneous treatment with both compounds more effectively inhibited the expression of key cell cycle regulatory proteins, including cyclin D1, E1, A2, and B1, than single-compound treatments (Figure 3D). However, the inhibitory effect of the combination of the two compounds on the expression levels of CDKs, such as CDK1, 2, and 4, was similar to that of ArcA alone (Figure 3D). These data suggest that the growth-inhibitory effect of the combination of berbamine and ArcA on GSCs may be implicated in the inactivation of CaMKIIγ-mediated cell cycle progression.

CaMKII activates cell proliferation and survival by upregulating multiple intracellular growth signaling pathways, such as Janus kinase/signal transducer and activator of transcription (JAK/STAT), phosphoinositide 3-kinase/protein kinase B (PI3K/AKT), and mitogen-activated protein kinase/extracellular signal-regulated kinase (MAPK/ERK) [44,47,48]. Thus, we assessed the effect of combined treatment with berbamine and ArcA on STAT3, AKT, and ERK1/2 signaling in U87MG-derived GSCs. As shown in Figure 3C, co-treatment with the two compounds strongly inhibited the total and phosphorylated protein expression of STAT3, AKT, and ERK1/2 in comparison with single-agent treatments. Collectively, these results demonstrate that the combination of berbamine and ArcA potently suppressed GSC growth by downregulating cell cycle regulatory proteins, including cyclins and CDKs, via inhibition of the CaMKIIγ-mediated STAT3/AKT/ERK1/2 signaling pathway.

### 2.4. Combined Treatment of Berbamine and ArcA Synergistically Suppresses Expression of GSC Markers

**Figure 4:**
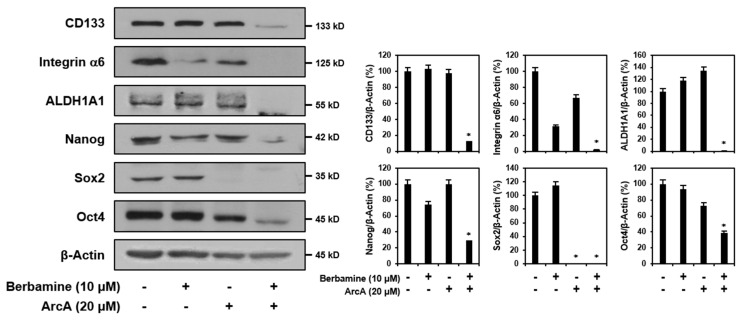
Combined treatment of berbamine and ArcA synergistically suppresses expression of GSC markers. U87MG-derived GSCs were treated with the indicated concentrations of berbamine and ArcA for 24 h. Protein levels were detected by Western blot analysis using specific antibodies and were further quantified by densitometry. β-Actin levels were used as an internal control. * *p* < 0.05 vs. the compound alone or the control.

The overexpression of several GSC markers has been implicated in the progression and recurrence of GBM [49,50,51]. Therefore, they are potential therapeutic targets for GBM. We investigated whether co-treatment with berbamine and ArcA affected the expression of key cancer stemness markers in GSCs. The main GSC markers include the transmembrane glycoprotein CD133; integrin α6, a regulator of stem cell–niche interactions; aldehyde dehydrogenase 1A1 (ALDH1A1), a detoxifying enzyme of hazardous aldehydes; and reprogramming transcription factors such as Nanog, Sox2, and Oct4, which are important for maintaining stem-like properties [52]. As shown in Figure 4, simultaneous treatment with the two compounds led to a significant reduction in the expression levels of CD133, integrin α6, ALDH1A1, Nanog, Sox2, and Oct4 compared to single-compound treatments. These results suggested that the synergistic anticancer effect of berbamine and ArcA on GSCs is related to the effective suppression of major GSC markers.

### 2.5. Synergistic Anticancer Effect of Berbamine and ArcA on GSCs Is Related to Dual Inhibition of CaMKIIγ and CDK4

**Figure 5:**
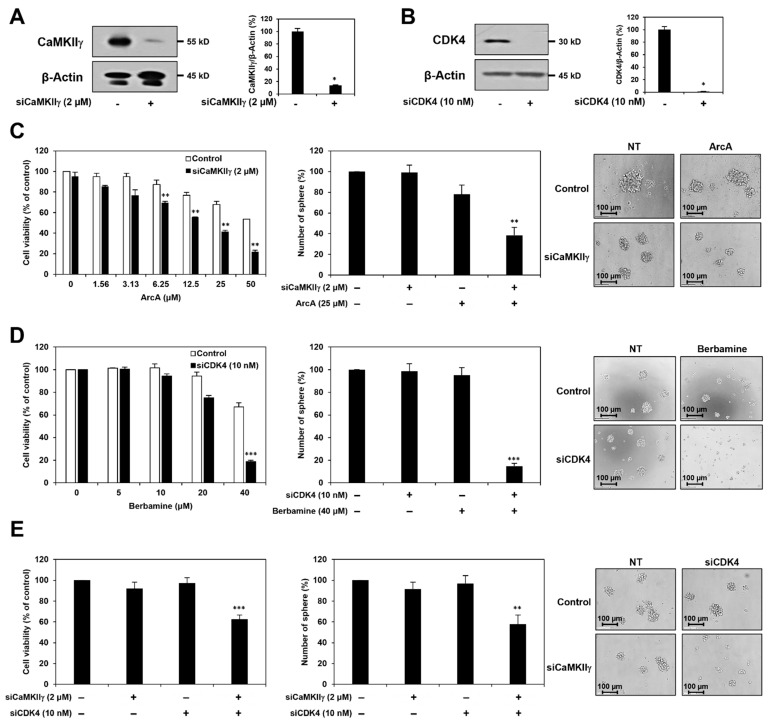
Synergistic anticancer effect of berbamine and ArcA on GSCs is related to dual inhibition of CaMKIIγ and CDK4. U87MG cells were transfected with either CaMKIIγ siRNA or CDK4 siRNA. Knockdown of (**A**) CaMKIIγ and (**B**) CDK4 genes was confirmed by Western blot analysis. Protein levels were detected by Western blot analysis using specific antibodies and were further quantified by densitometry. β-Actin levels were used as an internal control. * *p* < 0.05 vs. the control siRNA. Following genetic knockdown, U87MG-derived GSCs were treated with the indicated concentrations of (**C**) ArcA and (**D**) berbamine for 7 days. (**E**) Effect of simultaneous knockdown of CaMKIIγ and CDK4 genes on the cell viability and tumorsphere formation of U87MG-derived GSCs. (**C**–**E**) Cell viability was measured using the CellTiter-Glo^®^ luminescent assay system. The number of formed tumorspheres was counted under an optical microscope. ** *p* < 0.01, *** *p* < 0.001 vs. the compound alone or the single gene knockdown.

To elucidate whether the synergistic effect of berbamine and ArcA in eliminating GSCs was caused by the simultaneous inhibition of CaMKIIγ and CDK4, we performed genetic knockdown experiments using small interfering RNAs (siRNAs) targeting either CaMKIIγ or CDK4. U87MG cells were transfected with either CaMKIIγ-specific siRNA (siCaMKIIγ) or CDK4-specific siRNA (siCDK4). Silencing of each gene was confirmed by Western blot (Figure 5A,B). First, following CaMKIIγ knockdown, the U87MG-derived GSCs were treated with ArcA. As shown in Figure 5C, silencing of CaMKIIγ significantly increased the inhibitory effect of ArcA on cell viability and tumorsphere formation of U87MG-derived GSCs. Following CDK4 gene silencing, U87MG-derived GSCs were treated with berbamine. As shown in Figure 5D, CDK4 knockdown increased the chemosensitivity of U87MG-derived GSCs to berbamine. We further confirmed the effect of the concurrent knockdown of CaMKIIγ and CDK4 genes on GSCs. Synchronous silencing of both genes effectively suppressed cell viability and tumorsphere formation in U87MG-derived GSCs compared to silencing of each gene alone (Figure 5E). These results suggested that the synergistic anticancer effect of berbamine and ArcA on GSCs may result from the dual inhibition of CaMKIIγ and CDK4.

### 2.6. Combined Treatment of Berbamine and ArcA Potently Suppresses Tumor Growth Derived by GSCs In Vivo

**Figure 6:**
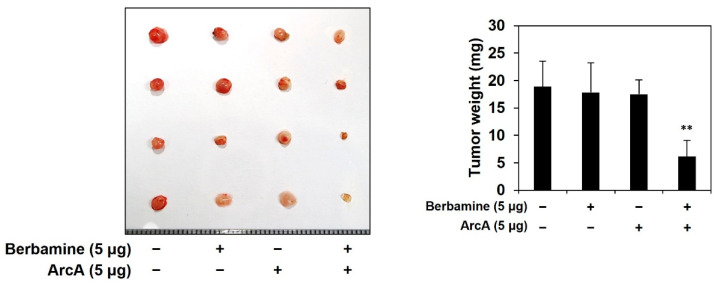
Combined treatment of berbamine and ArcA potently suppresses tumor growth derived by GSCs in vivo. Fertilized chick eggs were incubated in a humidified incubator at 37 °C. At embryonic day seven, U87MG-derived GSCs were mixed with ECM gel in the absence or presence of the indicated compounds (5 μg/egg) and were grafted onto the CAM surface. Seven days later, the CAMs were observed, the formed tumors were retrieved, and the tumor weight was calculated. ** *p* < 0.01 vs. the compound alone.

To further verify the effect of combined treatment with berbamine and ArcA on the tumorigenic potential of GSCs in vivo, we used a chick embryo chorioallantoic membrane (CAM) tumor model grafted with U87MG-derived GSCs. As shown in Figure 6, the tumor weight of the control group was 18.9 ± 4.6 mg, and those of berbamine and ArcA individual treatment were 17.8 ± 5.4 and 17.5 ± 2.6 mg, respectively. On the other hand, the tumor weight of co-treatment group of both compounds was 6.15 ± 3.0 mg, indicating that the combined administration markedly inhibited the GSC-derived tumor growth compared with the single-compound treatments. Therefore, these data demonstrated potent anticancer activity of the combination treatment of berbamine and ArcA in vivo.

## 3. Discussion

Numerous studies have demonstrated that GSCs play critical roles in GBM initiation, progression, invasiveness, resistance to therapies, and recurrence [3,5]. Therefore, the development of potential GSC-targeted therapies may improve therapeutic outcomes in GBM. Although several stemness markers and signaling pathways related to the increased malignant properties of GSCs have been characterized, exploring novel GSC biomarkers and effective therapeutic strategies is still challenging due to the various resistance mechanisms of GSCs to therapeutic agents [7,8,53].

CaMKIIγ is one of the four isoforms of CaMKII, which is a multifunctional serine/threonine-specific protein kinase [54]. Accumulating evidence has revealed that CaMKIIγ functions as an important molecular switch in several oncogenic signaling pathways, including nuclear factor kappa B (NF-κB), Wnt/β-catenin, ERK, AKT, and STAT3, and thus is closely implicated in the pathogenesis of cancer [55,56,57,58]. In addition, CaMKIIγ plays a crucial role in maintaining stem-like traits of CSCs, leading to tumor initiation, metastasis, drug resistance, and recurrence [23,24,59,60]. CaMKIIγ enhances the stemness and tumorigenicity of lung cancer cells by promoting AKT- and Wnt/β-catenin-mediated Oct4 expression [24,59]. CaMKIIγ is also overactivated in leukemia stem cells and upregulates Wnt/β-catenin, NF-κB, and STAT3 signaling, thereby promoting cell survival and self-renewal [23]. Moreover, berbamine, a CaMKIIγ inhibitor, inhibited the growth of leukemia stem cells by downregulating these signaling pathways [23]. Berbamine also suppressed the self-renewal ability of liver cancer stem cells, and genetic knockdown of CaMKIIγ recapitulated the effects of berbamine [60]. More recently, it has been demonstrated that CaMKIIγ is a promising therapeutic target to eliminate GSCs and inhibitors of CaMKIIγ suppress the stem-like features of GBM cells [27]. The CaMKIIγ inhibitors HBC and KN93 effectively blocked the self-renewal and metastatic capacities of GSCs by downregulating the CaM/CaMKIIγ/c-Met pathway. Furthermore, a new CaMKIIγ-targeted synthetic lethal therapy against GSCs was identified by performing high-throughput drug combination screening using CaMKIIγ inhibitors and a bioactive compound library in GSCs [25]. NK1R inhibitors, such as SR 140,333 and aprepitant, exhibited strong synthetic lethal interactions with CaMKIIγ inhibitors, including HBC, berbamine, and KN93, both in vitro and in vivo. This suggests the potential for a new combination therapy targeting CaMKIIγ and NK1R to eradicate GSCs. Further exploration of a novel anticancer treatment that displays a potent synergistic combination effect with a CaMKIIγ inhibitor in suppressing GSCs may contribute to overcoming chemoresistance and relapse of GBM.

The CDK4/6-cyclin D-Rb-E2F pathway plays a pivotal role in regulating cellular proliferation [29]. In proliferating cells, activated cyclin D–CDK4/6 complexes initiate Rb phosphorylation, thereby causing the functional inactivation of Rb. The subsequent release of the E2F transcription factor induces the expression of genes that are required to enter the S-phase for mitotic cell division. However, the CDK4/6-cyclin D-Rb-E2F pathway is hyperactivated in many human cancers, including GBM, resulting in uncontrolled tumor cell proliferation [31,36,61]. Notably, pathway-associated genes are altered in nearly 80% of the human gliomas [62,63]. Therefore, the inhibition of CDK4/6 may be an attractive therapeutic approach to block the initiation of GBM cell proliferation. Several selective CDK4/6 inhibitors such as palbociclib, ribociclib, and abemaciclib have been developed and widely used in preclinical and clinical trials for cancer treatment [36]. CDK4/6 inhibitors effectively arrest cancer cell proliferation in the G1-phase by inhibition of Rb phosphorylation [36]. In a rat intracranial GBM xenograft model, the combination of abemaciclib and TMZ additively increased survival time [64]. However, intrinsic or acquired resistance to CDK4/6 inhibitors has limited their application in cancer therapy [36]. A previous study revealed that CDK4 is a major self-renewal regulator of triple-negative breast CSCs, as well as a key mediator of resistance to chemotherapy [65]. Therefore, the discovery of a novel class of CDK4 inhibitors and the development of a new drug combination therapy with a CDK4 inhibitor may provide the advantage to effectively block GSC growth.

In the present study, we demonstrated for the first time that the combination of the CaMKIIγ inhibitor berbamine and CDK4 inhibitor ArcA synergistically increased GSC lethality in vitro and in vivo. Simultaneous treatment with both natural compounds markedly suppressed GSC viability and tumorsphere formation, and effectively inhibited tumor growth in a GSC-grafted CAM model, in comparison with the single-compound treatments. The synergistic anticancer effect of berbamine and ArcA on GSC growth results from the promotion of ROS- and calcium-dependent apoptosis through strong activation of the p53-mediated caspase cascade. Moreover, co-treatment with the two compounds potently suppressed the expression of several GSC markers, including CD133, integrin α6, ALDH1A1, Nanog, Sox2, and Oct4, which play central roles in GSC maintenance and drug resistance. Furthermore, the combination of berbamine and ArcA significantly downregulated the expression levels of cell cycle regulatory proteins by strongly inactivating the CaMKIIγ-mediated STAT3/AKT/ERK1/2 signaling pathway in GSCs. However, simultaneous treatment with both compounds more effectively inhibited the expression of cyclin D1, E1, A2, and B1 compared to single-compound treatments, whereas the inhibitory effect of the combination treatment on the expression of CDK1, 2, and 4 was similar to that of ArcA alone. These results suggest that the growth-inhibitory effect promoted by the combination of berbamine and ArcA in GSCs may be related to an increase in cell cycle arrest following synergistic inhibition of cyclin expression. Additionally, we demonstrated that the combined effect of berbamine and ArcA in eliminating GSCs resulted from the simultaneous inhibition of CaMKIIγ and CDK4 using siRNAs targeting either CaMKIIγ or CDK4. These data imply that suppression of CDK4 function by ArcA may increase the chemosensitivity of GSCs to the CaMKIIγ inhibitor berbamine. Taken together, our findings suggest that the combination treatment of berbamine and ArcA could be a potential therapeutic option to overcome GBM chemoresistance and recurrence by targeting GSCs.

## 4. Materials and Methods

### 4.1. Materials

Berbamine and ArcA were purchased from Sigma-Aldrich (Saint Louis, MO, USA) and Tocris (Bristol, UK), respectively. The compounds were dissolved in dimethyl sulfoxide (DMSO) at a final concentration of 100 mM. DMEM/F12 was purchased from HyClone (Marlborough, MA, USA). Accutase was obtained from EMD Millipore (Temecula, CA, USA). Epidermal growth factor (EGF) and basic fibroblast growth factor (bFGF) were purchased from Prospecbio (East Brunswick, NJ, USA). L-glutamine, B-27 serum-free supplement, and penicillin/streptomycin were purchased from Gibco (Grand Island, NY, USA). Heparin, 2′,7′-dichlorodihydrofluorescein diacetate (H_2_DCFDA), 4′,6-diamidino-2-phenylindole (DAPI), and ECM gel from Engelbreth-Holm-Swarm murine sarcoma (cat. no. E6909) were purchased from Sigma-Aldrich (Saint Louis, MO, USA). The ECM gel contains laminin as a major component, collagen type IV, heparin sulfate proteoglycan, entactin, and other minor components. The CellTiter-Glo^®^ 2.0 Cell Viability Assay (cat. no. G9241) and Muse^®^ Cell Cycle (cat. no. MCH100106) kits were purchased from Promega (Madison, WI, USA) and Luminex (Austin, TX, USA), respectively. Fluo-4 AM ester was purchased from Biotium (Hayward, CA, USA). Antibodies against p53 (cat. no. 9282), p21 Waf1/Cip1 (cat. no. 2947), cleaved caspase-9 (cat. no. 9501), cleaved caspase-3 (cat. no. 9661), PARP (cat. no. 9542), cyclin D1 (cat. no. 2922), cyclin E1 (cat. no. 4129), cyclin A2 (cat. no. 4656), cyclin B1 (cat. no. 12231), CDK1 (cat. no. 9116), CDK2 (cat. no. 2546), phospho-AKT (Ser473, cat. no. 4060), AKT (cat. no. 9272), phospho-STAT3 (Tyr705, cat. no. 9145), STAT3 (cat. no. 9139), phospho-ERK1/2 (Thr202/Tyr204, cat. no. 9101), ERK1/2 (cat. no. 9102), CD133 (cat. no. 64326), integrin α6 (cat. no. 3750), ALDH1A1 (cat. no. 12035), Nanog (cat. no. 3580), Sox2 (cat. no. 3579), Oct4 (cat. no. 2750), β-actin (cat. no. 4967), rabbit IgG (cat. no. 7074), and mouse IgG (cat. no. 7076) were purchased from Cell Signaling Technology (Danvers, MA, USA). Anti-phospho-CaMKIIγ (Thr287, cat. no. PA5-37833) and anti-CaMKIIγ (cat. no. PA5-29648) were obtained from Thermo Fisher Scientific (Rockford, IL, USA). Anti-CDK4 (cat. no. sc-70831) was obtained from Santa Cruz Biotechnology (Dallas, TX, USA).

### 4.2. GSC Culture

U87MG (KCLB No. 30014) and C6 (KCLB No. 10107) GBM cells were obtained from the Korean Cell Line Bank (Seoul, Republic of Korea). The identity of the GBM cell lines was confirmed by STR profiling as described previously [25]. GSC populations can be isolated from GBM cell lines and grown in a serum-free spheroid suspension culture [6,66]. To propagate GSCs, U87MG and C6 GBM cells were cultured in DMEM/F12 containing 1 × B-27, 5 μg/mL heparin, 2 mM L-glutamine, 20 ng/mL EGF, 20 ng/mL bFGF, and 1% penicillin/streptomycin as described previously [25,27]. Tumorspheres grown in the serum-free media were subcultured by dissociation using Accutase. The cells were maintained at 37 °C in a humidified CO_2_ incubator with 5% CO_2_ (Thermo Scientific, Vantaa, Finland).

### 4.3. Cell Viability Assay

U87MG- and C6-derived GSCs (3 × 10^3^ cells/well) were seeded in a 96-white-well culture plate using serum-free media and treated with the different concentrations of each compound for 7 days. Cell viability was determined using the CellTiter-Glo^®^ 2.0 Cell Viability Assay kit as described previously [25]. Luminescence was detected using a multimode microplate reader (BioTek, Inc., Winooski, VT, USA).

### 4.4. Tumorsphere Forming Assay

To evaluate the ability of a single GSC to grow into a non-adherent tumorsphere, U87MG- and C6-derived GSCs (3 × 10^3^ cells/well) were seeded in a 96-well culture plate using serum-free media and treated with the different concentrations of each compound for 7 days. The number of tumorspheres >80 µm in diameter was counted under an optical microscope (Olympus, Tokyo, Japan).

### 4.5. DAPI Staining

U87MG- and C6-derived GSCs (1 × 10^5^ cells/well) were seeded in a 12-well culture plate using serum-free media and treated with the indicated concentrations of each compound for 24 h. The cells were stained with 15 μg/mL DAPI for 30 min and then washed with phosphate-buffered saline (PBS). The stained nuclear morphology of the cells was observed under a fluorescence microscope (Optinity KI-2000F, Korea Lab Tech, Seong Nam, Republic of Korea).

### 4.6. Measurement of ROS

U87MG- and C6-derived GSCs (1 × 10^5^ cells/well) were seeded in a 12-well culture plate using serum-free media and treated with the indicated concentrations of each compound for 24 h. The cells were stained with 10 μM H_2_DCFDA for 30 min and then washed with PBS. The levels of intracellular ROS were observed under a fluorescence microscope (Optinity KI-2000F, Korea Lab Tech, Seong Nam, Republic of Korea) and quantified by measuring the DCF fluorescence intensity using ImageJ 1.5 software (NIH, Bethesda, MD, USA) in randomly selected four fields per group at a 200 × magnification.

### 4.7. Measurement of Calcium

U87MG- and C6-derived GSCs (1 × 10^5^ cells/well) were seeded in a 12-well culture plate using serum-free media and treated with the indicated concentrations of each compound for 24 h. The cells were stained with 15 μM Fluo-4 AM ester for 20 min and then washed with PBS. The calcium levels were observed under a fluorescence microscope (Optinity KI-2000F, Korea Lab Tech, Seong Nam, Republic of Korea) and quantified by measuring the Fluo-4 AM fluorescence intensity using ImageJ 1.5 software (NIH, Bethesda, MD, USA) in randomly selected four fields per group at a 200 × magnification.

### 4.8. Western Blot

The cell lysates were separated using 7.5–15% sodium dodecyl sulfate-polyacrylamide gel electrophoresis. The separated proteins were transferred to polyvinylidene difluoride membranes (EMD Millipore, Hayward, CA, USA). The blots were blocked with 5% skim milk in Tris-buffered saline with 1 × Tween-20 (TBST) at room temperature for 1 h and then immunolabeled with the primary antibodies (dilution 1:500–1:2000) overnight at 4 °C as described previously [24]. After washing with TBST, the membranes were incubated with horseradish peroxidase-conjugated secondary antibodies (dilution 1:2000) at room temperature for 1 h. Immunolabeling was detected using an enhanced chemiluminescence kit (Bio-Rad Laboratories, Hercules, CA, USA) according to the manufacturer’s instructions. The band density was analyzed using ImageJ 1.5 software (NIH, Bethesda, MD, USA). Expression levels were determined as the normalized ratio of each target protein to β-actin.

### 4.9. Cell Cycle Analysis

Distribution of cells in different stages of cell cycle was analyzed by flow cytometry using the Muse^®^ Cell Cycle kit. The kit utilizes propidium iodide (PI) staining to allow quantitative measurement of percentage of cells in the G0/G1, S, and G2/M phases. Briefly, U87MG- and C6-derived GSCs (2 × 10^5^ cells/well) were plated in a 6-well culture plate using serum-free media and treated with the indicated concentrations of each compound for 24 h. The cells were harvested, fixed with 70% ethanol, and stained with 200 µL of Muse^®^ Cell Cycle reagent as described previously [67]. Cell cycle distribution was analyzed using a Guava^®^ Muse^®^ Cell Analyzer (MuseSoft_V1.8.0.3; Luminex Corporation, Austin, TX, USA).

### 4.10. RNA Interference

To silence the expression of target genes, small interfering RNAs (siRNAs) for CaMKIIγ and CDK4 were obtained from Bioneer (Daejeon, Republic of Korea). The sequence of CaMKIIγ siRNA was as follows: (sense) 5′-GUAGAGUGCUUACGCAAAU-3′; (antisense) 5′-AUUUGCGUAAGCACUCUAC-3′. The sequence of CDK4 siRNA was as follows: (sense) 5′-CUGACUUUUAACCCACACA-3′; (antisense) 5′-UGUGUGGGUUAAAAGUCAG-3′. Nontargeting scrambled siRNA (cat. no. sc-37007, Santa Cruz Biotechnology) was used as a negative control. Cells were transfected with siRNAs using Lipofectamine^TM^ 2000 Reagent (Invitrogen, NY, USA), and the expression levels of target genes were detected by Western blot.

### 4.11. Chick Embryo Chorioallantoic Membrane (CAM) Assay

The effects of the compounds on GBM tumorigenesis in vivo were investigated using a modified CAM assay as described previously [25,67]. Briefly, fertilized chick eggs were incubated at 37 °C in a humidified egg incubator for 7 days, and the eggshell membrane was carefully peeled away. U87MG-derived GSCs (2 × 10^6^ cells/egg) were mixed with ECM gel (40 μL/egg) in the absence or presence of the compounds (5 μg/egg) and placed onto the CAM (8 eggs per group). After incubation for 7 days, the tumor formed on the CAM of live eggs (4–6 tumors per group) was retrieved, and the tumor weight was measured.

### 4.12. Statistical Analysis

The results are expressed as the mean ± standard deviation from at least three independent experiments. Statistical analysis was performed by analysis of variance with Tukey’s post hoc test using SPSS 9.0 software (SPSS Inc., Chicago, IL, USA). Statistical significance was set at *p* < 0.05.

## 5. Conclusions

In this study, we identified a novel CaMKIIγ-targeted combination therapy which utilizes berbamine and ArcA to eradicate GSCs. Combined treatment with berbamine and ArcA synergistically suppressed GSC growth, both in vitro and in vivo, by promoting caspase-dependent apoptosis and cell cycle arrest at the G0/G1 phase. Furthermore, the synergistic growth inhibitory effect of berbamine and ArcA on GSCs has been implicated in the potent downregulation of cell cycle regulatory proteins, including cyclins and CDKs, by inhibiting the CaMKIIγ-mediated STAT3/AKT/ERK1/2 signaling pathway. Moreover, simultaneous treatment with the two compounds markedly decreased the expression of key GSC markers such as CD133, integrin α6, ALDH1A1, Nanog, Sox2, and Oct4. In addition, a gene silencing study using siRNAs demonstrated that the synergistic anticancer effect of berbamine and ArcA on GSCs resulted from dual inhibition of CaMKIIγ and CDK4. These findings suggest a novel drug combination strategy consisting of berbamine and ArcA that simultaneously targets CaMKIIγ and CDK4 to effectively eradicate GSCs.

## Data Availability

The data that support the findings of this study are available from the corresponding author upon reasonable request.

## Supplementary Materials

The following supporting information can be downloaded at: https://www.mdpi.com/article/10.3390/molecules27227968/s1, Figure S1: Effect of berbamine and ArcA on the viability of GSCs; Figure S2: Effect of combined treatment with berbamine and ArcA on the proliferation of GSCs; Figure S3: Effect of combined treatment with berbamine and ArcA on the cell cycle in GSCs.
