# Supplementary material for: Synergistic Anticancer Effect of a Combination of Berbamine and Arcyriaflavin A against Glioblastoma Stem-like Cells
Source: Molecules. 2022 Nov 17;27(22):7968. doi: 10.3390/molecules27227968 (PMC9699626; doi:10.3390/molecules27227968)
Supplement: Supplementary file 1 [file molecules-27-07968-s001.zip › molecules-1996291-supplementary.pdf]

Article

Synergistic Anticancer Effect of a Combination of Berbamine and Arcyriaflavin A against Glioblastoma Stem-like Cells

Jang Mi Han [1] and Hye Jin Jung [1,2,3,*]

1 Department of Life Science and Biochemical Engineering, Graduate School, Sun Moon University, Asan 31460, Republic of Korea
2 Department of Pharmaceutical Engineering and Biotechnology, Sun Moon University, Asan 31460, Republic of Korea
3 Genome-Based BioIT Convergence Institute, Sun Moon University, Asan 31460, Republic of Korea
* Correspondence: poka96@sunmoon.ac.kr; Tel.: +82-41-530-2354; Fax: +82-41-530-2939

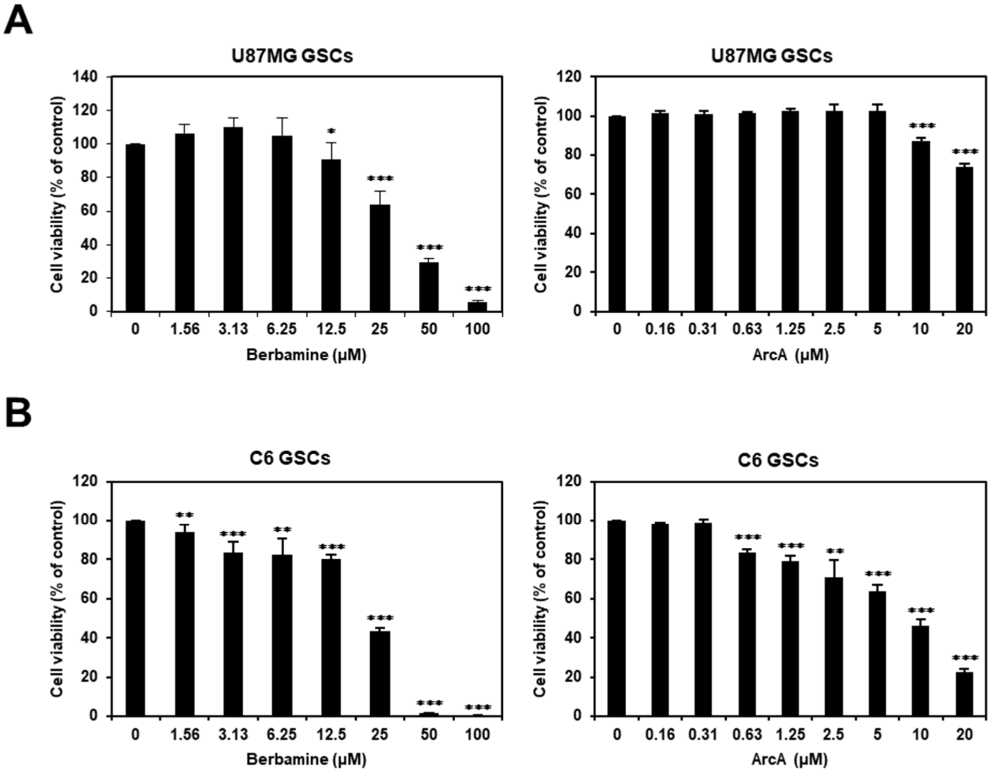

Figure S1. Effect of berbamine and ArcA on the viability of GSCs. (**A**) U87MG-derived GSCs were treated with the indicated concentrations of berbamine or ArcA for 7 days. (**B**) C6-derived GSCs were treated with the indicated concentrations of berbamine or ArcA for 7 days. Cell viability was measured using the CellTiter-Glo® luminescent assay system. * $p < 0.05$, ** $p < 0.01$, *** $p < 0.001$ vs. the control.

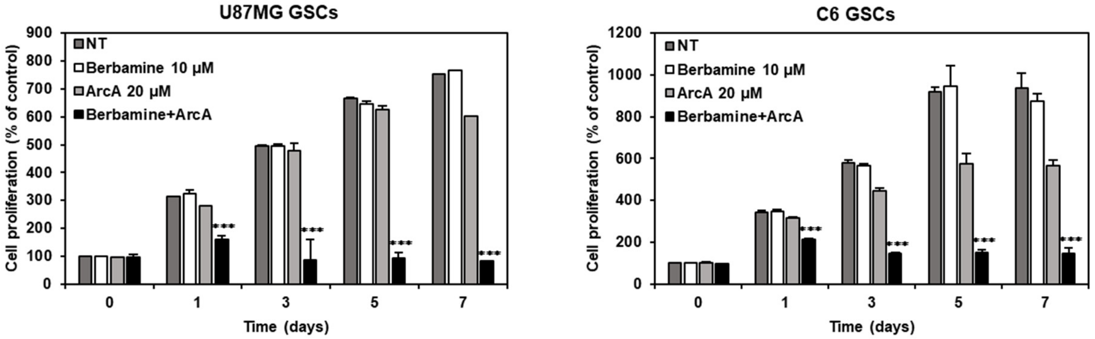

Figure S2. Effect of combined treatment with berbamine and ArcA on the proliferation of GSCs. U87MG- and C6-derived GSCs were treated with 10 μM berbamine and 20 μM ArcA for 7 days and cell proliferation was measured using the ATP-monitoring luminescence assay. *** $p < 0.001$ vs. the compound alone.

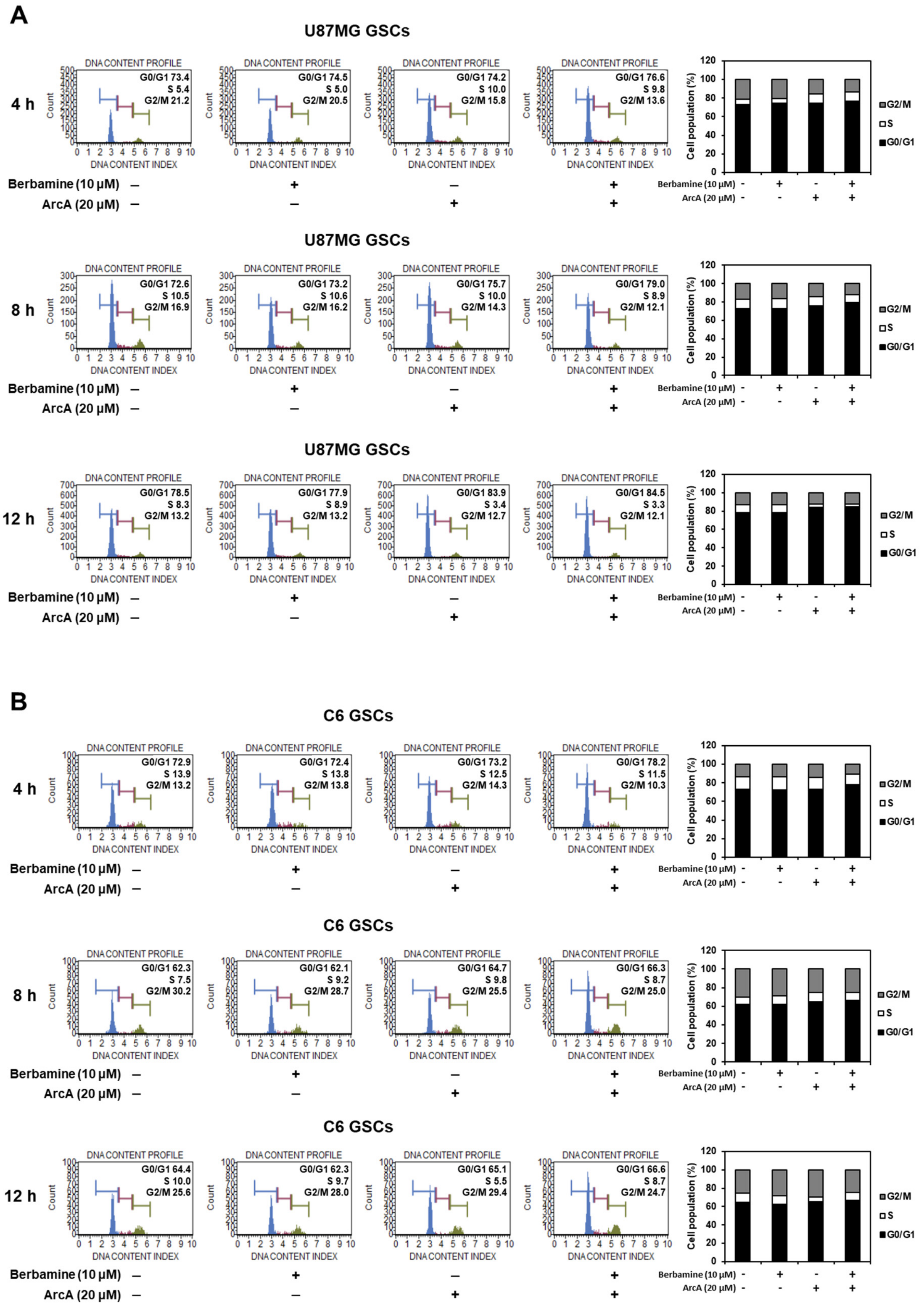

Figure S3. Effect of combined treatment with berbamine and ArcA on the cell cycle in GSCs. (**A**) U87MG- and (**B**) C6-derived GSCs were treated with berbamine (10 μM) and ArcA (20 μM) for 4, 8, and 12 h. Cell cycle distribution was detected using a Muse Cell Analyzer with Muse® Cell Cycle kit.
